# Axial length and keratometry characteristics of patients undergoing cataract surgery in Saudi Arabia

**DOI:** 10.25122/jml-2024-0122

**Published:** 2024-06

**Authors:** Ghada Aljuhani, Mona Alharbi, Rami Alsaidi, Abeer Alharbi

**Affiliations:** 1 Saudi Commission for Health Specialties, Madinah, Saudi Arabia; 2 Ophthalmology Department, Ohud Hospital, Madinah, Saudi Arabia; 3 Optometry Department, Ohud Hospital, Madinah, Saudi Arabia

**Keywords:** axial length, keratometry, cataract, IOL Master

## Abstract

Cataract surgery is one of the most successful surgical procedures, mainly due to the recent developments in surgical instruments and intraocular lens (IOL) measurements. Understanding the nature of axial length (AL) and keratometry readings (K) in patients with cataracts has significant implications for accurate postoperative IOL size selection. This study aimed to measure AL and K in patients undergoing cataract surgery in Saudi Arabia. This retrospective study included patients who underwent cataract surgery in Madinah, Saudi Arabia. The medical records of adult patients between May 2022 and May 2023 were reviewed, and those with a history of retinal detachment, refractive surgery, or trauma were excluded. The AL and K readings were obtained from the patient’s IOL master results. A total of 691 eyes from 451 patients were included in the analysis. The mean age was 64.54 years old. Most of the patients were women (55%). The mean AL, K1, and K2 values were 23.27, 43.42, and 44.69, respectively. Linear regression analysis was used to measure the relationship between AL and K, revealing an inverse relationship in our findings. As AL increased by one unit, the mean K was estimated to decrease by 0.548, with a 95% confidence interval. Our results demonstrated an inverse relationship between AL and K, implying that when AL increases, K decreases, or the corneal curvature becomes flat. Further studies are needed to investigate the biomechanical mechanisms underlying this relationship.

## INTRODUCTION

Globally, age-related cataract is the leading cause of reversible blindness [1-4]. In Saudi Arabia, the estimated prevalence of cataracts is 30.75%, similar to the global prevalence of 30% [5]. Cataract surgery is the standard treatment for lens opacities worldwide, and, with recent advancements in ophthalmology, it is among the most effective surgical procedures in medicine [6].

Following cataract surgery, ophthalmologists typically implant primary or secondary intraocular lenses (IOLs) of the appropriate size. Accurate ocular biometrics are crucial for precise IOL sizing. The IOL Master, a non-contact tool for ocular measurement, uses partial coherence interferometry and has been found to be more accurate than other biometric devices [7]. The average regular axial length (AL) in emmetropic adults ranges from 22.33 to 22.99 mm [8]. The mean keratometry value (K) in adult patients with cataract using IOL Master 500 was 44.42 [9].

The anterior surface of the cornea's curvature is estimated using keratometry measurements, which is essential for determining the refractive power of the eye. The distance between the anterior surface of the cornea and the retina is known as the axial length [10]. There is a significant correlation between AL and K under typical physiological circumstances. Generally, a steeper cornea correlates with a shorter AL, while a flatter cornea is linked to an increase in AL. This connection is crucial because it affects the eye's refractive state and determines the IOL power needed for cataract surgery [11].

Previous studies have investigated the importance of understanding the biometric characteristics of patients undergoing cataract surgery. One study demonstrated that accurate AL and K measurements are essential for predicting postoperative refractive outcomes [12]. Another study emphasized the impact of biometric variations on IOL power calculation accuracy [13]. Despite these findings, there is limited data on the biometric profiles of patients with cataract in Saudi Arabia.

This study aimed to evaluate the axial length and keratometry characteristics of a cohort of patients who underwent cataract surgery in Madinah, Saudi Arabia. By analyzing the IOL Master’s results and their associations with various patient demographics and ocular parameters, we intend to provide valuable insights into the biometric profiles of patients with cataracts in this region. To the best of our knowledge, this is the first study to investigate the characteristics of AL and K in patients with cataracts in Saudi Arabia using IOL Master’s results. The findings of this research could serve as a basis for future studies and contribute to decision-making in clinical practice, promoting an improved understanding of the unique ocular biometric traits among the population in our region. The importance of conducting this research lies in the lack of localized data that can imply clinical practice specific to the Saudi population.

## MATERIAL AND METHODS

This retrospective observational study used the IOL Master to obtain data from 691 eyes from 451 patients who underwent cataract surgery between May 2022 and May 2023. Medical documents of eligible patients were reviewed to obtain demographic information, including age, sex, and laterality of the eye. Preoperative ocular biometric measurements were performed for each eye, including AL and K. Patients with histories of retinal detachment, trauma, or refractive surgery were excluded from the analysis.

The IOL Master is a non-contact optical biometer that utilizes partial coherence interferometry to accurately measure AL and anterior chamber depth (ACD). Additionally, this device includes an automated keratometer for assessing corneal curvature. Measurements were conducted by experienced technicians who adhered to the manufacturer’s recommended protocols. During the measurements, patients were instructed to fixate on a target with their fellow eye. A minimum of five correct AL and K readings were obtained for each eye, and the average value was used for analysis (Carl Zeiss, IOL MASTER, Technology V.7.3)

The data were then entered into Microsoft Excel version 16.66.1 and exported to SPSS version 29 for data analysis. Descriptive statistics were calculated for the ocular biometric parameters (AL and K), including the mean, standard deviation, minimum, and maximum. Linear regression was performed to assess the relationship between AL and K, and bivariate correlation analysis was used to define the relationship between age and ocular parameters. An independent sample *t*-test was used to compare the mean values of the ocular biometric parameters between male and female patients. Paired *t*-tests were used to compare the mean values of the right and left eyes. Statistical significance was set at *P* <0.05.

## RESULTS

A total of 691 eyes from 451 patients were evaluated. Most of our patients were women (250, 55.4%). The mean age was 64.54 years old, with a minimum of 19 years minimum, and a maximum of 93 years. Three hundred and forty-eight measurements were obtained from the right eye (50%). The mean, minimum, and maximum values of AL, K1, and K2 are listed in Table 1. The mean astigmatism value was -1.27.

**Table 1:** Descriptive data for AL, K1, and K2

	AL	K1	K2
Mean	23.27	43.42	44.69
Minimum	19.61	35.01	38.57
Maximum	27.72	48.63	54.97

AL, axial length; K, keratometry value

**Figure 1:**
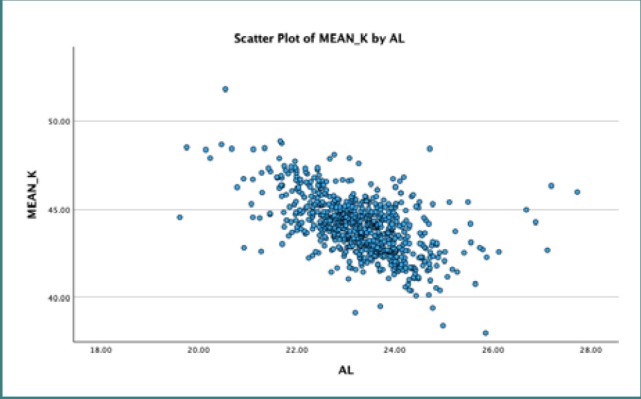
Scatter plot illustrating the inverse relationship between axial length and mean K reading

**Figure 2:**
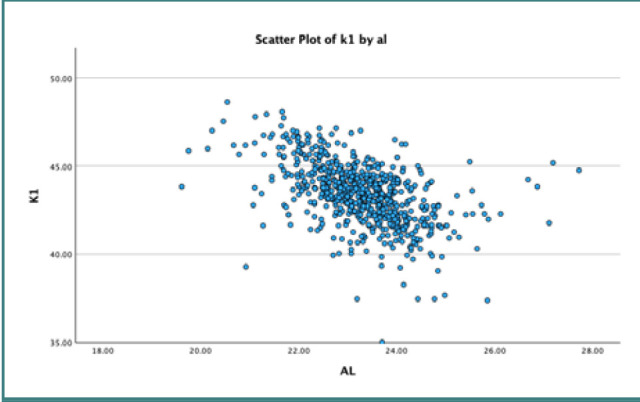
Scatter plot illustrating the inverse relationship between axial length and K1 reading

**Figure 3:**
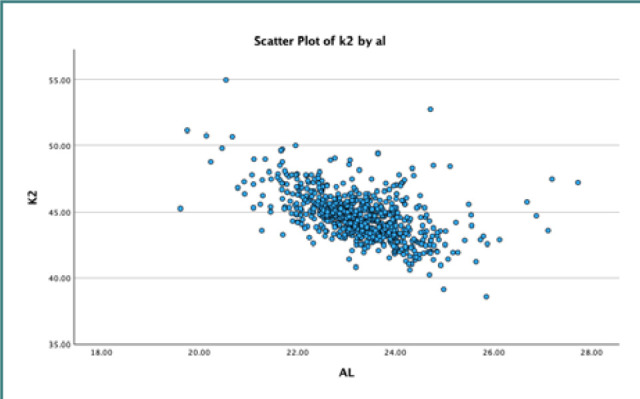
Scatter plot illustrating the inverse relationship between axial length and K2 reading

We applied linear regression analysis with a 95% confidence interval to explore the association between AL and mean K. Our results indicated a statistically significant inverse relationship between AL and mean K, as evidenced by a negative regression coefficient of -0.548, with a standard deviation of 0.054. These analyses are further detailed in Table 2. Figures 1–3 represent scatter plots illustrating the relationship between AL and K.

**Table 2:** The relationship between AL and K

	Coefficient	Standard deviation
AL and the mean K	-.548	0.054
AL and K1	-.514	0.018
AL and K2	-.522	0.018

AL, axial length; K, keratometry value

### Relationship between ocular biometrics and age

**Figure 4:**
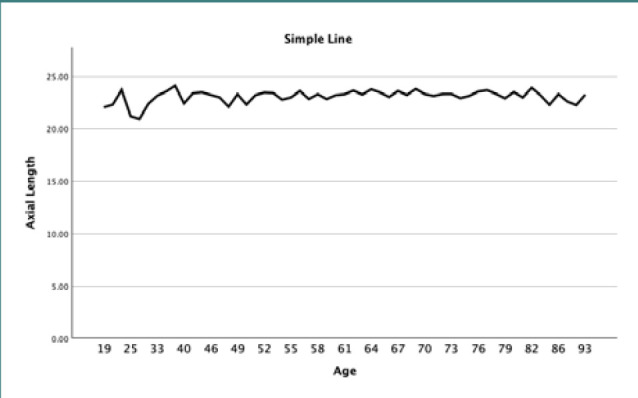
Line plot illustrating the relationship between axial length and age

**Figure 5:**
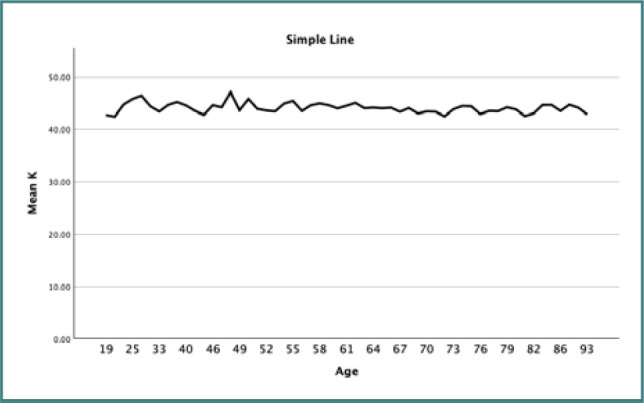
Line plot illustrating the relationship between mean keratometry reading and age

Bivariate correlation analysis was used to evaluate the relationship between AL and age. The correlation coefficient for the relationship between AL and age was 0.085, which had a positive correlation, indicating that with an increase in age, AL also tended to increase. Conversely, the relationship between K and age displayed a correlation coefficient of -0.088, indicating a similarly weak negative correlation, with K values decreasing as age increases. However, both relationships were statistically insignificant, with *P* values above 0.05 (Table 3). Figures 4 and 5 present line plots that visually depict these relationships between age and the measurements of AL and K, respectively.

**Table 3:** Relationship between ocular biometrics and age

	coefficient (r)	*P* value
AL and age	0.085	0.070
K and age	- 0.088	0.062

AL, axial length; K, keratometry value

### Relationship between ocular biometrics and gender

An independent samples *t*-test was used to determine the relationship between ocular biometrics and gender. An independent samples *t*-test was used to determine the relationship between ocular biometrics and gender.

**Figure 6:**
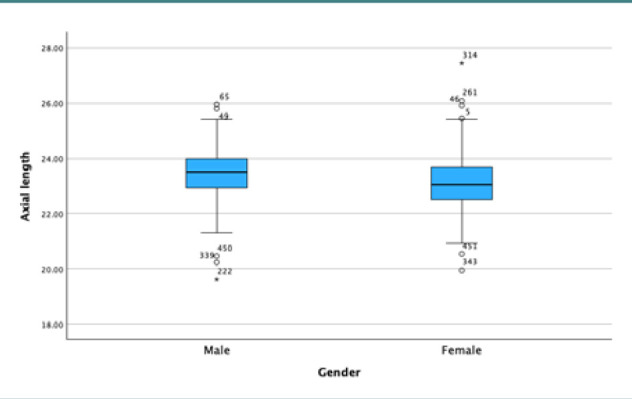
Box plot illustrating the relationship between axial length and gender

**Figure 7:**
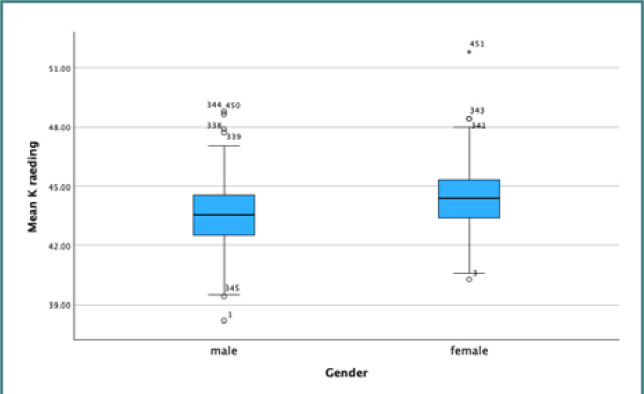
Box plot illustrating the relationship between mean keratometry value and gender

Our results demonstrated a positive *t*-value of 4.334 for the relationship between AL and gender and a negative *t*-value of -5.498 for mean K. However, the *P* values for axial length and K were above 0.05, indicating no statistically significant difference between the two groups (Table 4). Figures 6 and 7 display box plots illustrating the relationship between gender and AL and K.

**Table 4:** Relationship between ocular biometrics and gender

	t statistic	P value
AL and gender	4.334	0.701
K and gender	-5.498	0.521

AL, axial length; K, keratometry value

### Interocular differences

To assess the differences in AL and K between the eyes, we employed a paired sample *t*-test. Two-sided *P* values are displayed in Table 5. No statistically significant differences were noted between the two eyes.

**Table 5:** Interocular differences

Variable name	Mean differences	*P* value	Standard deviation
AL	.027	.432	.034
K1	.005	.927	.894
K2	.21	.728	.950

AL, axial length; K, keratometry value

## DISCUSSION

Our study evaluated the axial length and keratometry characteristics in a cohort of patients undergoing cataract surgery in Madinah, Saudi Arabia. The sample consisted of 691 eyes from 451 patients, with 55% of the participants being women and an average age of approximately 64 years. The mean values of AL, K1, and K2 were 23.27 mm, 43.42 D, and 44.69 D, respectively.

We observed a significant inverse relationship between AL and K values. Specifically, as AL increased, K decreased, a finding consistent with previous studies [11,14,15]. This relationship can be attributed to physiological changes: during infancy, as the axial length increases rapidly, the cornea and lens tend to flatten, pushing the refractive state toward emmetropia. In later life, the axial length tends to stabilize or decrease slightly, which correlates with a slight steepening of the cornea.

Despite our results showing no significant relationship between age and AL or K, literature reviews and past studies present mixed findings. While some studies report a decrease in AL with aging due to lens thickening and axial shortening, others found no significant effect [16]. The lack of significant findings in our study might be attributed to the specific age distribution of our participants or other confounding factors, such as body length and education, which needed to be controlled for in this study.

The mean K values (43.42 D for K1 and 44.69 D for K2) identified in this study align with previous findings [17]. This consistency suggests that the keratometry values measured by the IOL Master 500 are reliable across different populations. However, variations in K values can also arise due to differences in the measurement methods and demographic factors like ethnicity and geographical location.

Our study investigated the relationship between gender and biometric parameters. We found a positive correlation between AL and gender, as well as between mean K and sex, although these correlations were not statistically significant. Previous research has consistently demonstrated significant gender differences in K values, typically attributed to hormonal influences and anatomical variations [18]. The lack of significant findings in our study may be attributable to factors such as sample size limitations or specific characteristics of the population under study. Interocular differences in AL, K1, and K2 between the two eyes were minimal and not statistically significant. This contrasts with studies on large cohorts and twin studies, which have noted significant differences between right and left eyes, often with the right eye being longer [19]. These discrepancies could be due to the random variability in our sample or differences in the study designs and populations.

Our findings provide valuable insights into the biometric profiles of patients with cataracts in Madinah, Saudi Arabia. The observed inverse relationship between AL and K, the consistency with previous studies on K values, and the lack of significant age-related changes highlight the importance of considering population-specific factors when evaluating ocular biometrics. Future research should include a more diverse age range and control for additional confounding variables to understand these relationships better. Our study is a foundational step toward improving IOL power calculations and surgical outcomes in this region.

### Limitations

The statistical analytical tests used in this study did not establish causality or consider other potential risk factors.

## CONCLUSION

Our study concluded that AL and K in patients undergoing cataract surgery were similar to those of other studies in different population groups and were inversely correlated. We observed no statistically significant interocular differences in AL measurements or K values. Further studies that replicate or investigate the same relationship are needed to confirm the nature of the connections between the different variables examined in our study.
